# Childhood Body Fat Patterns and Obesity Prevalence in Kazakhstan

**DOI:** 10.1002/osp4.70024

**Published:** 2024-11-26

**Authors:** Shynar Abdrakhmanova, Altyn Aringazina, Zhanar Kalmakova, Laura Utemissova, Mirjam Heinen, Marta Buoncristiano, Julianne Williams, Kremlin Wickramasinghe, Mohammed T Hudda

**Affiliations:** ^1^ The National Center of Public Healthcare of the Ministry of Health of the Republic of Kazakhstan Almaty Kazakhstan; ^2^ KMU “Kazakhstan School of Public Health” Almaty Kazakhstan; ^3^ Almaty Management University AlmaU Almaty Kazakhstan; ^4^ Caspian University Almaty Kazakhstan; ^5^ Kyzylorda High Medical College Kyzylorda Kazakhstan; ^6^ World Health Organization Country Office Astana Kazakhstan; ^7^ Special Initiative on NCDs and Innovation WHO Regional Office for Europe Copenhagen Denmark; ^8^ Department of Population Health Dasman Diabetes Institute Kuwait City Kuwait

**Keywords:** body composition, fat mass prediction equation, pediatric obesity

## Abstract

**Background:**

In Kazakhstan the pediatric population levels of obesity based on fat mass (FM) assessment are currently unknown. The present work aimed to assess average childhood FM levels and the prevalence of high levels of adiposity (based upon FM levels).

**Methods:**

Cross‐sectional data from 2015 to 2020 nationally representative Childhood obesity surveillance initiative and 2022 regional surveys were used for this study of children aged 8 years (*n* = 4770) and 9 years (*n* = 3863). Childhood FM assessment was made using a validated prediction model using height, weight, age, sex and ethnicity. Average levels of FM, fat mass percent (FM%) and the prevalence of overfat and obesity were estimated.

**Results:**

Amongst 8‐year‐olds, the population average FM% was 32.3% (95% CI: 31.7%–32.8%) for boys and 35.2% (95% CI: 34.8–35.6) for girls (2015) and 32.7% (95% CI: 32.3–33.1) for boys and 35.1% (95% CI: 34.7–35.5) for girls in 2020. The Almaty region had the average FM% 32.7% (95% CI: 32.1–33.2) and 34.8% (95% CI: 34.3–35.4) for boys and girls respectively in 2022. The similar pattern was observed for 9 year old children.

**Conclusions:**

The present study reveals high FM% levels in primary school age children from Kazakhstan across study years. Understanding patterns of FM levels is important for preventing and addressing childhood obesity.

## 1 Introduction

Childhood obesity is a global public health threat due to its short and long term adverse health effects [1, 2]. In order to understand obesity trends, inform and support programs and interventions on childhood obesity prevention and management, in 2007 the WHO Regional Office for Europe established the WHO European Childhood Obesity Surveillance Initiative (COSI) [3, 4].

In the most recent round of COSI data collection, conducted between 2018 and 2020 in 33 countries, the large differences between countries persist in overweight (including obesity) rates with higher prevalence in the Southern Europe to lower rates in Central Asia [4]. In 2020 the overall prevalence of overweight and obesity among 8 year old children (boys and girls combined) in Kazakhstan was 21.1% and 7.1% respectively [4, 5]. This reported obesity prevalence amongst Kazakh children was similar to that reported by a previous study where the prevalence among 5–19 year olds was 6.5% in 2016 [6].

However, such estimates of childhood overweight and obesity prevalence have been based upon body mass index (BMI; weight/height^2^). Although BMI remains the most commonly used, widely accepted, and practical measure of obesity in both children and adults, particularly for surveillance [2, 4, 7, 8], BMI suffers from several limitations [8, 9, 10, 11, 12, 13]. Crucially, as it is a weight‐based marker, it cannot distinguish between fat mass (FM) and fat free (lean) mass (FFM), which have been shown to vary for the same level of BMI in childhood [8, 9, 13, 14, 15]. Additionally, childhood BMI has been shown to provide biased estimates in ethnic minority childhood populations [16, 17] and remains correlated with childhood height due to the poor standardization for height (using a height‐squared term) [9, 18, 19].

Assessment of childhood adiposity based on more direct techniques, rather than indirect markers such as BMI, would represent an important advance in the field of childhood adiposity. It would allow to obtain more accurate assessments of body composition and crucially of FM levels, which have been shown to be the portion of childhood weight associated with long‐term type 2 diabetes, cardiovascular risks [2, 20, 21]. While there are several techniques to assess body composition in childhood, such as bioelectrical impedance (BIA) and dual‐energy X‐ray absorptiometry (DXA), these techniques are not suitable for population‐level due to their associated costs and may lack precision [22, 23, 24]. However, the availability of an accurate equation for the prediction of childhood FM and FFM, based on readily available measurements of weight, height, age, sex, and ethnicity, allows for the estimation of childhood body composition without the need for more complex forms of assessment [9, 25, 26]. This equation has been extensively validated in several childhood populations globally and has been demonstrated to produce accurate estimates of childhood FM and FFM [25].

Currently, no population‐level information pertaining to body composition levels for children from Kazakhstan or the Central Asian region is available, and thus quantifying these levels and patterns over time are extremely important as part of the efforts to reduce childhood obesity levels globally.

Therefore, in these nationally representative and regional studies, the assessment focused on average childhood FM levels and the prevalence of high levels of adiposity (based upon FM levels) in Kazakhstan between 2015 and 2020 and in Almaty city region (2022).

## 2 Materials and Methods

### 2.1 Study Population and Sample Design

The current study is affiliated with the WHO European COSI survey. COSI Kazakhstan started in 2015 in response to the need for establishing obesity surveillance among primary school children [3, 4, 5, 27]. For Kazakhstan, COSI 2015 and 2020 were national representative surveys conducted in 7 and 17 regions respectively, while COSI 2022 was a regional survey conducted in Almaty city only. Kazakhstan COSI 2015 targeted 9 year old children, while in 2020 and 2022 the surveys targeted 8 year old Kazakhstani children [4, 27].

The study sample included children, aged 8–9 years in the second, third and fourth grades of public schools in Kazakhstan, who took part in the COSI national surveys conducted in 2015 and 2020 or the regional study conducted in 2022.

The Republic of Kazakhstan is an upper middle‐income Central Asian country. At the beginning of 2023, the child population (0–15 year olds) accounted for 6.2 million (31.2%) of the 19.8 million people. Total population ethnic composition consists of 70.7% Kazakh, 15.2% Russian, 14.1% other ethnic origins [28].

A two staged stratified cluster sampling design with schools as a primary sampling units and classes as the secondary sampling units was used. In order to reach the number of target age children (2800 per age group), at each of the randomly selected schools, two grades were sampled and defined the second sampling stage. In 2015, one class from third grade and one class from fourth grade were selected. The sample was nationally representative, drawn from 7 regions. In 2020, the target classes were from second and third grades. National representative sample of schools and classes was selected from 17 regions of the country. In 2015 and 2020 stratification variables were region and degree of urbanization. In 2022, the survey was implemented only in one urban region, the country's biggest megapolis. Children from second and third grades were selected based on a representative sample of city schools, the sample was stratified by city rayons. More information about the COSI study population, sampling design and data collection procedures are presented elsewhere [3, 4, 5, 27].

### 2.2 Data Collection Procedures

Data collection was performed by trained examiners according to a standard protocol developed by WHO Regional Office for Europe and Member States [3, 5, 29]. The WHO COSI study protocol was approved by the International Ethical Guidelines for Biomedical Research Involving Human Subjects, and all procedures were also approved by local Ethics Committees from National Center for problems of healthy lifestyle development (2015), National Center of Public healthcare (Protocol №2 dated September 11, 2020), Kazakhstan's Medical University “Kazakhstan School of Public Health” (Protocol №IRB‐A146 dated July 3, 2021). Parents of children were informed about the study procedures. A passive approach for parental inform consent was used.

### 2.3 Anthropometric Measurements

Children's weight and height were measured by trained interviewers using WHO standardized techniques [3, 29]. Measurements were taken in light clothes where appropriate, without shoes. Body weight was measured using Seca 813 digital scales to the nearest 0.1 kg, and height was measured using Seca 213 stadiometers to the nearest 1 mm. Child weight was adjusted to the type of clothes worn at the measurements. Parents or caregivers completed a proxy questionnaire on child's social‐demographic, family, nutrition and physical activity characteristics.

### 2.4 Statistical Analysis

STATA version 17 was used for all analyses. The equation used to predict childhood FFM and FM from height, weight, age, sex and ethnicity for this study was originally developed in a dataset of 2375 UK children aged 4–15 years who had reference standard deuterium dilution measures of body composition [8]. Extensive external validation of the equation amongst UK children [8, 26] as well as children from a further 19 childhood settings globally [25], demonstrated the model's high levels of accuracy at predicting childhood FFM and FM. As part of the external validation study, the original UK‐based equation was re‐calibrated for use in several other childhood settings including Russian Federation [25]. Given the geographic location and similarities between the two populations, the re‐calibrated equation for use in Russian Federation (shown below) was applied within the two Kazakhstan COSI and one Almaty regional survey datasets to predict FFM (and thus FM by subtraction from weight measurements).

$${\text{Fat}\;\text{Mass}}_{\text{Russia}-\text{equation}}=\text{Weight}-\text{exponential}\left(0.3073\times {\text{height}}^{2}-10.0155\times {\text{weight}}^{-1}+0.004571\times \text{weight}+0.01408\times \text{BA}-0.06509\times \text{SA}-0.02624\times \text{AO}-0.01745\times \text{Other}-0.9180\times \ln\left(\text{age}\right)+0.6488{\times \text{age}}^{0.5}+0.04723\times \text{male}+2.6897\right)$$

Fat mass percent (FM%; fat mass/weight × 100) was also calculated. Complete information on the ethnic origins of the children was not available, however given that almost all children residing in Kazakhstan will likely be of Other Asian origins [28, 30], this assumption was made for the estimation of FM and FFM, as was also the case for the previous external validation study in Russian Federation [25].

Within the analysis sample, key variables of weight, height and level of urbanization were summarized in terms of their mean values and standard deviations (weight and height) or *n* (%) for urbanization within each of the three surveys, by sex and age group. To take account of the survey‐based study design of the COSI survey, weighted data analyses were carried out in order to draw inferences for the whole population based on the results from the surveyed children and to produce unbiased estimates. Sampling weights were estimated by the WHO Regional Office for Europe, which adopted a common approach that considered the sampling design applied in each country taking part in the specific COSI survey round [3].

Within each survey round, sex‐specific population‐level means (and 95% confidence intervals) for 8‐ and 9‐year‐olds were estimated for FM (kg) and FM%. The Wald test was used, at the 5% level, to test for statistical differences in average FM and FM% levels by survey year and sex. Due to differences in the relationship between FM and height at each age, within each survey round and between boys and girls, it was not feasible to standardize FM to form a height‐independent fat mass index (FMI) which was consistent across subgroups. Therefore, the FM patterns were explored over time and by sex on the original scale (i.e., FM in kg). Also, a sensitivity analysis was performed to assess the impact of childhood height on these results by splitting the samples into tertiles of height and re‐estimated the age‐ and sex‐specific population‐level mean FM, by height groups, within each survey round.

In addition, the sex‐ and age‐specific prevalence (and associated 95% confidence intervals) of high childhood FM levels (referred to here as “overfat” and “obesity”) was also estimated for each survey round using the 85th (“overfat”) and 95th (“obesity”) centiles from the US Centers for Disease Control and Prevention reference curves [31] due to similarities in the distribution of FM% between this population (Supporting Information S1: Tables S1 and S2) and those used to derive the US centiles. Statistical tests for the difference in proportions were conducted to formally examine differences by survey year and sex using the 5% level.

## 3 Results

### 3.1 Descriptive Statistics

This study included a total of 4770 children aged 8 years and 3863 children aged 9 years, distributed as follows. 2015 national study: 8 years‐old (*N* = 1843) and 9 year‐old (*N* = 2918) children; 2020 national study: 8 years‐old (*N* = 2927) and 9 year‐old (*N* = 945) children. Almaty regional study 2022 included *N* = 1063 and *N* = 630 children aged 8 and 9 years respectively. Descriptive results of the analysis sample as mean levels and standard deviation for height and weight by sex and survey year are presented in the Table 1. The tables also contain number and percentage of urban children in the sample. The mean height of 8 and 9 year old boys at the national level was 1.30 m in 2015 and 2020, and 1.34 m in 2015 and 2020, respectively. The mean weight of 8 year old boys was 28.1 and 28.2 kg, whereas the mean weight of 9 year old boys was 30.4 and 30.9 kg in 2015 and 2020, respectively. The mean height of 8‐ and 9‐year‐old girls was 1.29 and 1.34 m in 2015 and 2020, respectively. For 8‐year‐old girls the mean weight was 27.1 kg in 2015, 27.2 kg in 2020, whereas for 9 year old girls—29.9 and 29.7 kg in 2015 and 2020, respectively (Table 1).

**TABLE 1:** Descriptive statistics of analysis sample by sex and survey year.

	Sample mean (SD)/*n* (%)					
	Boys			Girls		
Survey year	2015	2020	2022	2015	2020	2022
Age 8 years						
*N*	912	1483	534	931	1444	529
Number of regions	7	17	1	7	17	1
Urbanization	514 (56%)	846 (57%)	534 (100%)	560 (60%)	817 (57%)	529 (100%)
Height (m)	1.30 (0.06)	1.30 (0.06)	1.32 (0.06)	1.29 (0.06)	1.29 (0.06)	1.31 (0.06)
Weight (kg)	28.1 (5.4)	28.2 (5.9)	28.9 (5.6)	27.1 (5.8)	27.2 (5.6)	27.7 (6.2)
Age 9 years						
*N*	1491	510	327	1427	435	303
Number of regions	7	17	1	7	17	1
Urbanization	1088 (73%)	307 (60%)	327 (100%)	1050 (74%)	264 (61%)	303 (100%)
Height (m)	1.34 (0.07)	1.34 (0.06)	1.37 (0.06)	1.34 (0.08)	1.34 (0.07)	1.36 (0.06)
Weight (kg)	30.4 (6.1)	30.9 (7.0)	32.2 (7.0)	29.9 (6.3)	29.7 (6.4)	30.5 (6.7)

*Note:* 2015 and 2020 contain data from more than 1 region in Kazakhstan, whereas 2022 was conducted in the Almaty region solely.

In Almaty sample (2022 study year) the mean height of 8‐ and 9‐year‐old boys was 1.32 and 1.37 m, respectively, and the mean weight was 28.9 and 32.2 kg, respectively. Eight‐ and nine‐year‐old girls had the mean height 1.31 and 1.36 m, and the mean weight 27.7 and 30.5 kg, respectively (Table 1).

### 3.2 Population‐Level Estimates of Fat Mass and Fat Mass Percentage in 2015, 2020, and 2022

#### 3.2.1 Nationally Representative Results (2015 and 2020)

The sex‐, survey year‐ and age‐specific estimates of the population‐level mean values of FM and FM% are summarized in Table 2.

**TABLE 2:** Population‐level estimated means of fat mass (kg) and fat mass percent for Kazakhstan children by sex, survey year, and age.

	Population mean (95% CI)			
	Boys		Girls	
	2015	2020	2015	2020
Age 8 years				
Fat mass (kg)	9.1 (8.7–9.6)	9.5 (9.2–9.7)	9.8 (9.5–10.1)	9.7 (9.5–9.9)
Fat mass percent	32.3 (31.7–32.8)	32.7 (32.3–33.1)	35.2 (34.8–35.6)	35.1 (34.7–35.5)
Age 9 years				
Fat mass (kg)	9.8 (9.4–10.2)	10.6 (10.1–11.0)	11.0 (10.7–11.3)	10.8 (10.4–11.2)
Fat mass percent	32.2 (31.7–32.7)	33.4 (32.8–34.0)	35.6 (35.1–36.0)	35.5 (35.1–36.0)

Amongst 8 year old Kazakhstani boys in 2015, the average FM was estimated to be 9.1 kg (95% CI: 8.7–9.6 kg) with an average FM% of 32.3% (95% CI: 31.7%–32.8%). The average levels for FM and FM% were similar in 2020: was 9.5 kg (95% CI: 9.2–9.7 kg) and 32.7% (95% CI: 32.3%–33.2%) respectively (Table 2). Amongst 9 year old boys in 2015, the average FM was estimated to be 9.8 kg (95% CI: 9.4–10.2 kg) with an average FM% of 32.2% (95% CI: 31.7%–32.7%). The average levels marginally increased in 2020 to 10.6 kg (95% CI: 10.1–11.0 kg; *P*_Wald_ = 0.01) and 33.4% (95% CI: 32.8%–34.0%; *P*_Wald_ = 0.004) for both FM and FM% respectively (Table 2).

Amongst 8 and 9 year old Kazakhstani girls, no difference in average levels of FM and FM% between 2015 and 2020 was observed (*P*_Wald_ > 0.05) (Table 2). However, average FM and FM% levels were higher amongst girls than for boys (*P*_Wald_ < 0.001) with the exception of FM in 2020 where no statistical difference was observed.

Sensitivity analyses exploring the impact of height on the above main results, which did not factor height into the analysis, found consistent patterns across survey rounds and by sex when the results were re‐analyzed by tertiles of height (Supporting Information S1: Table S3).

#### 3.2.2 Almaty Regional Results (2022)

In Almaty city in 8‐ and 9‐year boys the mean level of FM was 9.7 kg (95% CI: 9.3–10.0 kg) and 10.9 kg (95% CI 10.4–11.3 kg), respectively. The average FM% was 32.7% (95% CI: 32.1%–33.2%) in 8‐year‐olds and 33.1% (95% CI: 32.5%–33.6%) in 9 year old boys (Supporting Information S1: Table S4). Average levels amongst girls were similar for FM to those observed for boys, but there was difference in the average FM% levels compared to boys (*P*_Wald_ < 0.001) (Supporting Information S1: Table S4).

### 3.3 Prevalence of Overfat and Obesity

#### 3.3.1 National Results (2015 and 2022)

Table 3 presents the prevalence of high adiposity levels of Kazakhstani primary school children. For boys, the prevalence of overfat (including obesity) amongst 8‐year‐olds in 2015 was 15.3% (95% CI: 11.5%–20.2%). There was borderline increase (*P*_test of proportions_ = 0.05) in the prevalence by 2020%, to 20.4% (95% CI: 17.8%–23.3%). The prevalence of overfat (including obesity) amongst 9‐year‐old boys was 14.7% (95% CI: 11.7%–18.2%) in 2015 and increased (*P*_test of proportions_ = 0.03) to 20.5% (95% CI: 16.8%–24.8%) by 2020.

**TABLE 3:** Population‐level estimated prevalence of overfat (including obesity) and obesity among children by sex, survey year, and age (%).

	Boys		Girls	
	2015	2020	2015	2020
8 Year‐olds				
Overfat (inc obesity)	15.3 (11.5–20.2)	20.4 (17.8–23.3)	18.0 (14.4–22.2)	17.9 (15.2–21.0)
Obesity	5.0 (3.4–7.3)	6.3 (5.0–8.0)	5.6 (3.8–8.1)	5.5 (4.3–7.0)
9 Year‐olds				
Overfat (inc obesity)	14.7 (11.7–18.2)	20.5 (16.8–24.8)	16.9 (13.6–20.8)	17.1 (13.2–21.8)
Obesity	3.2 (2.0–5.1)	8.1 (5.8–11.2)	7.3 (4.9–10.7)	4.0 (2.2–7.1)

*Note:* Sex‐specific overfat and obesity cut‐offs defined by Ogden et al. 2011 as ≥ 85th and ≥ 95th centiles, respectively (www.cdc.gov/nchs/data/nhsr/nhsr043.pdf).

For girls, the prevalence of overfat (including obesity) between 2015 and 2020 remained consistent amongst both 8‐year‐olds (Table 3; 2015: 18.0%, 2020: 17.9%) and 9 year olds (Table 3; 2015: 16.9%, 2020: 17.1%). No difference was observed in the prevalence of obesity alone for either 8‐ or 9‐year‐olds (*P*_test of proportions_ > 0.05) (Table 3).

#### 3.3.2 Almaty Regional Results (2022)

The Almaty regional prevalence of overfat (including obesity) and for obesity alone are presented in Supporting Information S1: Table S5. In 2022 the prevalence of overfat (including obesity) amongst 8‐year‐old boys was 19.3% (95% CI: 15.1%–24.4%) and amongst 9‐year‐old boys was 23.8% (95% CI: 19.4%–28.7%).

There was no significant difference in the prevalence amongst girls compared to boys for 8‐year olds (*P*_test of proportions_ = 0.29), but there was a difference amongst 9‐year‐olds (*P*_test of proportions_ = 0.01) were the prevalence amongst girls was estimated to be 13.6% (95%CI: 9.1%–19.8%). In Almaty city, the prevalence of obesity was similar amongst boys and girls at age 8 years (boys: 5.7%, girls: 5.5%) and 9 years (boys: 6.3%, girls: 4.7%) (Supporting Information S1: Table S5).

## 4 Discussion

The main findings on this study highlight the extremely high levels of average FM amongst Kazakhstani children aged 8 and 9 years, with more than 30% of their body weight being attributed to FM. These high childhood body composition FM%, remained markedly high at national level across 2015, 2020 study years and did not increase among girls and 8‐year‐old boys. However, amongst 9‐year‐old boys FM% level increased over time from 2015 to 2020. The latest (2022) childhood FM and FM% data also demonstrated high adiposity levels in urban childhood population of one region. The average FM% levels were higher amongst girls than for boys both in national and regional studies.

The results of the present study reveal high levels of the prevalence of 8‐ and 9‐year‐old children living with overfat (including obesity). The prevalence remains constant across the study years at the population‐level (2015 and 2020) for girls and 8‐year‐old boys, while an increase in prevalence of overfat was found for 9 year old boys. The prevalence of obesity for 8‐ and 9‐year‐old boys and girls had similar patterns to those observed for overfat. In Almaty region the gender difference was observed with lower prevalence of 9‐year‐old girls living with overfat (including obesity) compared to prevalence of the same age boys.

The WHO defines obesity by “excessive fat deposits that can impair health,” which can influence an individual's quality of life. FM based assessments allow us to focus directly on the portion of weight which can impair health rather than weight as a whole. Furthermore, the recently updated framework by the European Association for the Study of Obesity for the diagnosis, staging and management of obesity in adults [32] and follow‐up correspondence regarding children [33] suggest that a change is needed to the way we assess obesity. The paper presents an advancement in understanding childhood adiposity by focusing on fat mass (FM)‐based metrics rather than the BMI‐based classifications that have predominantly characterized Kazakhstan's primary school children in previous published data. The study introduces several novel aspects providing a more nuanced perspective on childhood adiposity, highlighted below.

For the first time population average FM levels for 8 and 9 year old children in Kazakhstan were presented. FM‐based prevalence of overfat and obesity are more precise indication of adiposity and potentially reveal different patterns and prevalence rates compared to those based on BMI. Finally, application of FM (and FM%) to identify at‐risk individuals may reveal individuals that BMI‐based data misclassified as “healthy”.

The findings of this study demonstrating that children in Kazakhstan have high level of body fat is particularly relevant in light of recent studies, estimating the comparable FM and FM% levels in neighboring country. A recent study of children from Russian Federation reported the average level of deuterium dilution assessed FM to be 11.3 kg [25], which is similar to the average FM levels observed in our study of slightly younger and shorter children aged 9 years. In addition, a further study reported average FM% amongst Russian children under 13 years of age of 30.4% and 34.3% amongst boys and girls respectively, which were similar to levels presented in our study [34]. Another study from the Almaty region of Kazakhstan reported FM% levels among 160 children aged 9–10 years, however they presented average FM% levels separately for children with normal BMI levels and for those classified as living with obesity using BMI [35]. Among children with normal weight, average FM% was 17.7% and 19.4% for boys and girls respectively, while among children with obesity average FM% was 31.9% and 32.2% respectively [35]. This is problematic as it is known that within an individual, FM% levels can vary markedly for a given BMI [14], and thus these results are not comparable with those of the current study.

FM and FM% levels in 8–9 year old Kazakhstani boys and girls in our study are much higher than in 7 year old children in UK from the Millennium Cohort Study where the mean FM was 5.4 and 5.9 kg in boys and girls respectively and mean FM% was 20.2% and 22.3% in boys and girls respectively [36]. Similarly, in a birth cohort of 10‐year‐old children of Copenhagen average FM% levels was 23.5% in boys and 27.1% in girls [20] which were lower than population level FM% means in our study.

An average FM% levels in body composition of Kazakhstani children at 2015, 2020 studied years found to be much higher than among pediatric population in US (among 8 year old boys FM% 27.6% and among girls 31.4%) [31], in UK (among 8 years boys FM% 17.0% and among girls 21.2%) [37], in Portugal (among 10 year old boys FM% 29.3% and among girls 30.4%) [38], in Türkiye (among 8 year old boys FM% 20.9% and among girls 22.6%) [39], in India (among 8 year old boys FM% 22.7% and among girls FM% 25.3%) [40], in Korea (among 12 year old boys FM% 19.1% and among girls 26.3%) [41], and in China (among 9–19 year old boys, BF% 19.9% and among girls 28.1%) [42].

The difference in the body fat values across population is likely due to difference in ethnic and geographical characteristics, age groups, and FM assessment method used [9, 43]. A number of studies established that children of Asian origin had higher values of body fat compared to children of White Caucasian, Hispanic and Black origin [41, 44, 45, 46].

## 5 Strengths and Limitations of the Study

The present study has several strengths. Due to the study design and nationally representative survey in 2015 and 2020 the average population level means and prevalence were well estimated. The good sample size allows to make estimates of average values with good precision. Moreover, a standardized methodology across all study years enables to compare national surveys and present regional data. Validated equation allows to accurately assess FM levels where this was previously not possible.

Several study limitations should be acknowledged. First, the 2022 study sample is not nationally representative with only the Almaty regional study. Therefore, a direct comparison was not applicable between the 2015, 2020, and 2022 studies. However, 2015 and 2020 surveys were compared and 2022 data was presented separately.

Second, as FM hasn't been widely used as an indicator for childhood obesity, it might be difficult to draw comparisons with other countries, or over time in a single country. However, the availability of this open‐access equation to accurately estimate childhood body composition in historic and contemporary studies using only measurements which are readily available both, provides an exciting opportunity for this to be looked at in other settings and over time.

Third, due to the lack of childhood cut‐offs to define high levels of FM% from Kazakhstan, and even from the wider region, the US based cut‐offs were utilized [31]. While the left‐hand side of FM% distribution in the current study showed differences to that of the US study [36], the right‐hand side of the distributions were similar and thus, were used to define high levels of adiposity. Alternative methods for defining high levels of adiposity, particularly in this region, are required.

In addition, it was unable to standardize FM for height as the height‐power needed to standardize FM was not consistent across study years, sex or age (data not presented). However, a sensitivity analysis exploring the impact of height on our results did not show any material differences in the findings across tertiles of childhood height.

## 6 Conclusions and Implication for Further Research

The present study reveals high FM% levels in primary school age children at the national and regional studies. Across the study years (2015 and 2020) FM% levels remained high and constant, except for an increase in FM% amongst 9‐year‐old boys in 2020.

The pattern of prevalence of primary school children who were living with overfat and obesity were stable across national surveys (2015, 2020) with the exception of 9 year old boys for whom the prevalence increased by 2020.

The use of equation for the prediction of childhood FFM and FM with routinely collected anthropometric data allowed to build accurate understanding of patterns of childhood FM levels in Kazakhstan. High level of FM in studied pediatric population suggests that assessment of body fatness is important for public health surveillance and pediatrics health screening with appropriate cut‐offs and/or cut off values of body FM specific to child ethnic and geographical background.

Population based patterns of childhood FM explored in the present study identified trends in obesity rates and populations at higher health risk. This knowledge is essential for developing regional and global effective interventions, guiding public health policies, and ultimately reducing the burden of obesity‐related health issues in children.

High FM levels in Kazakhstan compared to children populations from other geographical locations requires further research. Childhood FM is important as it has been shown to be the portion of weight which is more strongly related to type 2 diabetes risk in adults [18], therefore there is a need to shift toward FM assessment in clinical and population‐level surveillance. In addition, studies should be conducted to estimate FM distribution among Kazakhstan children in transition into adulthood in terms of health risks. Further work is needed to develop childhood FM cut‐offs which are indicative of obesity related disease risk in short‐ and long‐term instead of using centile based cut‐offs.

## Author Contributions

S.A. L.U. and Z.K. organized study data collection in 2015, 2020 and S.A. and A.A. organized study data collections in 2022. K.W., J.W., M.H. and M.B. made substantial contribution to the COSI study protocol. S.A. and M.T.H. conceived the idea for the study. M.T.H. analyzed the data, S.A. and M.T.H. drafted the first version of the manuscript. S.A., M.T.H., A.A., L.U., Z.K., M.H., M.B., J.W. and K.W. were involved in writing, manuscript revisions, and approved the final version of the manuscript.

## Conflicts of Interest

The authors declare no conflicts of interest.

## Supporting information

Supporting Information S1
