# Supplementary material for: Childhood Body Fat Patterns and Obesity Prevalence in Kazakhstan
Source: Obes Sci Pract. 2024 Nov 26;10(6):e70024. doi: 10.1002/osp4.70024 (PMC11589656; doi:10.1002/osp4.70024)
Supplement: Supplementary file 1 — Supporting Information S1 [file OSP4-10-e70024-s001.docx]

**Childhood body fat patterns and obesity prevalence in Kazakhstan**

Shynar Abdrakhmanova^1,2^, Altyn Aringazina^3,4^, Zhanar Kalmakova^1^_,_ Laura Utemissova^5^, Mirjam Heinen^6^, Marta Buoncristiano^6^, Julianne Williams^6^, Kremlin Wickramasinghe^6^, Mohammed T Hudda^7^

^1^ The National Center of Public Healthcare of the Ministry of Health of the Republic of Kazakhstan, Almaty, Astana, Kazakhstan

^2^ KMU “Kazakhstan School of Public Health”, Almaty, Kazakhstan

^3^ Almaty Management  University AlmaU, Almaty, Kazakhstan

^4^ Caspian University, Almaty, Kazakhstan

^5^ World Health Organization Country Office, Astana, Kazakhstan

^6^ Special Initiative on NCDs and Innovation, WHO Regional Office for Europe, Copenhagen, Denmark

^7^ Department of Population Health, Dasman Diabetes Institute, Kuwait City, Kuwait

**Corresponding author information:**

Mohammed T Hudda

Tel: +965 2224 2999 (ext 2517)

Email: [mohammed.hudda@dasmaninstitute.org](mailto:mohammed.hudda@dasmaninstitute.org)

Department of Population Health

Dasman Diabetes Institute

Kuwait City

Kuwait

**Supplementary Table S1: Percentiles of fat mass percent amongst boys, by age**

|  | **Percentiles of FM%** | | | | | |
| --- | --- | --- | --- | --- | --- | --- |
|  | 5 | 25 | 50 | 75 | 85 | 95 |
| Age 8 years |  |  |  |  |  |  |
| 2015 | 26.7 | 29.7 | 31.5 | 34.0 | 36.3 | 41.2 |
| 2020 | 25.7 | 29.4 | 32.0 | 35.3 | 37.4 | 42.7 |
| 2022 | 26.4 | 29.3 | 32.2 | 35.0 | 37.6 | 42.4 |
| Age 9 years |  |  |  |  |  |  |
| 2015 | 26.4 | 29.2 | 31.7 | 34.3 | 37.0 | 40.7 |
| 2020 | 25.7 | 29.9 | 32.6 | 35.9 | 38.7 | 44.3 |
| 2022 | 25.9 | 29.4 | 32.2 | 36.5 | 38.4 | 43.5 |

Footnote: 2015 and 2020 contain data from more than 1 region in Kazakhstan, whereas 2022 was conducted in the Almaty region solely and thus should not be compared directly to the 2015 and 2020 data.

**Supplementary Table S2: Percentiles of fat mass percent amongst girls, by age**

|  | **Percentiles of FM%** | | | | | |
| --- | --- | --- | --- | --- | --- | --- |
|  | 5 | 25 | 50 | 75 | 85 | 95 |
| Age 8 years |  |  |  |  |  |  |
| 2015 | 28.9 | 32.3 | 34.3 | 37.5 | 39.7 | 43.9 |
| 2020 | 28.4 | 32.0 | 34.6 | 37.8 | 39.9 | 43.9 |
| 2022 | 28.7 | 31.7 | 34.0 | 37.2 | 39.1 | 43.8 |
| Age 9 years |  |  |  |  |  |  |
| 2015 | 28.8 | 32.1 | 34.8 | 38.1 | 40.9 | 45.3 |
| 2020 | 28.3 | 32.3 | 35.2 | 38.4 | 40.8 | 43.5 |
| 2022 | 27.7 | 31.6 | 34.0 | 37.6 | 39.8 | 44.1 |

Footnote: 2015 and 2020 contain data from more than 1 region in Kazakhstan, whereas 2022 was conducted in the Almaty region solely and thus should not be compared directly to the 2015 and 2020 data.

**Supplementary Table S3: Population-level estimated means of fat mass (kg) and fat mass percent for Kazakhstan children by height tertiles, sex, survey year, and age**

|  | **Population mean (95% CI)** | | | | | |
| --- | --- | --- | --- | --- | --- | --- |
|  | **Height Group 1** | | **Height Group 2** | | **Height Group 3** | |
|  | 2015 | 2020 | 2015 | 2020 | 2015 | 2020 |
| **Boys** |  |  |  |  |  |  |
| Age 8 years |  |  |  |  |  |  |
| Fat Mass (kg) | 7.5 (7.2 - 7.8) | 7.9 (7.6 - 8.2) | 9.1 (8.6 - 9.5) | 9.3 (9.0 - 9.7) | 11.0 (10.4 - 11.5) | 11.3 (10.8 - 11.8) |
| Fat Mass Percent | 31.1 (30.5 - 31.6) | 31.8 (31.2 - 32.3) | 32.3 (31.6 - 33.1) | 32.6 (32.0 - 33.2) | 33.5 (32.8 - 34.3) | 33.8 (33.2 - 34.5) |
|  |  |  |  |  |  |  |
| Age 9 years |  |  |  |  |  |  |
| Fat Mass (kg) | 8.4 (7.9 - 8.9) | 8.8 (8.4 - 9.3) | 9.5 (9.2 - 9.9) | 10.3 (9.5 - 11.1) | 11.3 (10.8 - 11.8) | 13.3 (12.3 - 14.3) |
| Fat Mass Percent | 31.6 (30.7 - 32.6) | 32.4 (31.6 - 33.1) | 31.9 (31.3 - 32.5) | 33.0 (31.8 - 34.2) | 33.3 (32.5 - 34.0) | 35.2 (34.1 - 36.3) |
|  |  |  |  |  |  |  |
| **Girls** |  |  |  |  |  |  |
| Age 8 years |  |  |  |  |  |  |
| Fat Mass (kg) | 8.1 (7.8 - 8.4) | 8.1 (7.9 - 8.4) | 9.8 (9.3 - 10.3) | 9.6 (9.3 - 9.9) | 11.9 (11.1 - 12.6) | 11.5 (11.0 - 12.0) |
| Fat Mass Percent | 34.1 (33.6 - 34.6) | 34.2 (33.6 - 34.7) | 35.4 (34.6 - 36.1) | 35.1 (34.6 - 35.6) | 36.4 (35.3 - 37.6) | 36.1 (35.4 - 36.8) |
|  |  |  |  |  |  |  |
| Age 9 years |  |  |  |  |  |  |
| Fat Mass (kg) | 9.0 (8.6 - 9.4) | 8.1 (7.9 - 8.4) | 10.8 (10.4 - 11.2) | 10.7 (10.2 - 11.2) | 13.1 (12.3 - 13.9) | 13.2 (12.3 - 14.0) |
| Fat Mass Percent | 34.6 (33.7 - 35.5) | 34.6 (33.9 - 35.3) | 35.6 (35.0 - 36.2) | 35.6 (34.8 - 36.4) | 36.5 (35.6 - 37.5) | 36.7 (35.7 - 37.8) |

**Supplementary Table S4: 2022 Almaty Regional Results: Estimated means of fat mass (kg) and fat mass percent by sex and age**

|  | **Regional mean (95% CI)** | |
| --- | --- | --- |
|  | **Boys** | **Girls** |
| Age 8 years |  |  |
| Fat Mass (kg) | 9.7 (9.3 - 10.0) | 9.8 (9.4 - 10.2) |
| Fat Mass Percent | 32.7 (32.1 - 33.2) | 34.8 (34.3 - 35.4) |
|  |  |  |
| Age 9 years |  |  |
| Fat Mass (kg) | 10.9 (10.4 - 11.3) | 10.8 (10.2 - 11.5) |
| Fat Mass Percent | 33.1 (32.5 - 33.6) | 34.8 (34.1 - 35.5) |

**Table S5: 2022 Almaty Regional Results: Estimated prevalence of overfat (including obesity) and obesity among children, by sex and age, (%)**

|  | **Boys** | **Girls** |
| --- | --- | --- |
| 8 Year-olds |  |  |
| Overfat (inc obesity) | 19.3 (15.1 - 24.4) | 16.0 (12.6 - 20.1) |
| Obesity | 5.7 (3.9 - 8.2) | 5.5 (4.0 - 7.5) |
|  |  |  |
| 9 Year-olds |  |  |
| Overfat (inc obesity) | 23.8 (19.4 - 28.7) | 13.6 (9.1 - 19.8) |
| Obesity | 6.3 (3.9 – 10.2) | 4.7 (2.8 - 7.9) |

Footnote: Sex-specific overfat and obesity cut-offs defined by Ogden et al. 2011 as ≥85^th^ and ≥95^th^ centiles, respectively ([www.cdc.gov/nchs/data/nhsr/nhsr043.pdf](http://www.cdc.gov/nchs/data/nhsr/nhsr043.pdf)).
